# Body-Mounted Robotic System for MRI-Guided Shoulder Arthrography: Cadaver and Clinical Workflow Studies

**DOI:** 10.3389/frobt.2021.667121

**Published:** 2021-05-10

**Authors:** Niravkumar Patel, Jiawen Yan, Gang Li, Reza Monfaredi, Lukasz Priba, Helen Donald-Simpson, Joyce Joy, Andrew Dennison, Andreas Melzer, Karun Sharma, Iulian Iordachita, Kevin Cleary

**Affiliations:** ^1^ LCSR, Johns Hopkins University, Baltimore, MD, United States; ^2^ Children’s National Health System, Washington, DC, United States; ^3^ Institute for Medical Science and Technology, University of Dundee, Dundee, United Kingdom; ^4^ Institute for Computer Aided Surgery, University Leipzig, Leipzig, Germany

**Keywords:** shoulder arthrography, patient-mounted robot, MRI, Thiel, preclinical

## Abstract

This paper presents an intraoperative MRI-guided, patient-mounted robotic system for shoulder arthrography procedures in pediatric patients. The robot is designed to be compact and lightweight and is constructed with nonmagnetic materials for MRI safety. Our goal is to transform the current two-step arthrography procedure (CT/x-ray-guided needle insertion followed by diagnostic MRI) into a streamlined single-step ionizing radiation-free procedure under MRI guidance. The MR-conditional robot was evaluated in a Thiel embalmed cadaver study and healthy volunteer studies. The robot was attached to the shoulder using straps and ten locations in the shoulder joint space were selected as targets. For the first target, contrast agent (saline) was injected to complete the clinical workflow. After each targeting attempt, a confirmation scan was acquired to analyze the needle placement accuracy. During the volunteer studies, a more comfortable and ergonomic shoulder brace was used, and the complete clinical workflow was followed to measure the total procedure time. In the cadaver study, the needle was successfully placed in the shoulder joint space in all the targeting attempts with translational and rotational accuracy of 2.07 ± 1.22 mm and 1.46 ± 1.06 degrees, respectively. The total time for the entire procedure was 94 min and the average time for each targeting attempt was 20 min in the cadaver study, while the average time for the entire workflow for the volunteer studies was 36 min. No image quality degradation due to the presence of the robot was detected. This Thiel-embalmed cadaver study along with the clinical workflow studies on human volunteers demonstrated the feasibility of using an MR-conditional, patient-mounted robotic system for MRI-guided shoulder arthrography procedure. Future work will be focused on moving the technology to clinical practice.

## 1 Introduction

Magnetic resonance (MR) arthrography of the glenohumeral joint enables better assessment of the rotator cuff (Flannigan et al., 1990; Hodler et al., 1992) and glenoid labrum tears (Symanski et al., 2017). During MR arthrography procedure, a gadolinium-based contrast agent is injected into the intra-articular space of the glenohumeral joint (Jbara et al., 2005) and then MR images are acquired for diagnosis. The contrast agent is injected under ultrasound (Ćićak et al., 1992; Valls and Melloni, 1997), x-ray/fluoroscopy (Farmer and Hughes, 2002; Jacobson et al., 2003) or CT (Binkert et al., 2003; Hauth et al., 2016) guidance. MR arthrography with contrast agent injection under MRI guidance has also been explored (Hilfiker et al., 1999; Petersilge et al., 1997; Fritz et al., 2009; Soh et al., 2008; Wybranski et al., 2017). Except for the MRI-guided contrast agent injection approach, these approaches require two different imaging modalities, which are not usually available in adjacent rooms and require two separate procedures. The patient may also need to wait between procedures, which could cause the contrast agent to wash out, resulting in deteriorated image quality. The injection procedures under fluoroscopy/CT guidance result in exposure to radiation, which should be avoided, especially in pediatric patients. Although arthrography under x-ray is a relatively simple procedure, its diagnostic value is limited, and it involves ionizing radiation. MRI arthrography, however, is technically more difficult but it provides much more diagnostic information than x-ray and it avoids ionizing radiation. Performing arthrography under intraoperative MRI guidance could completely eliminate the exposure to radiation and streamline the procedure.

MR images have exquisite soft tissue contrast without any ionizing radiation, making it an ideal choice for image-guided percutaneous procedures for pediatric patients. However, performing the procedure under MRI guidance poses challenges such as high magnetic field strength (1.5–3 T), confined space (60–70 cm bore size) and limited ergonomic access to the patient. Also, performing manual needle insertion requires the clinician to place the needle while looking at the images, which can result in inaccurate needle placement and multiple insertions. Performing the needle insertion using a MRI-compatible robotic device could enable more accurate needle placement in the intra-articular joint space without requiring multiple needle passes.

The MR environment limits the choices of materials that can be safely used inside the scanner bore. Due to its high magnetic field strength, only plastics and non-magnetic metals can be used in the MR environment. In recent years, robotic devices using such materials and nontraditional actuators based on piezoelectric, hydraulic or pneumatic principles have been studied for various needle-based percutaneous interventions. Most MRI-guided robotic systems can be categorized into two categories: 1) table-mounted and 2) body-mounted. Table-mounted robots are rigidly attached to the scanner bed and their heft and bulk make them suitable only for certain percutaneous interventions such as prostate biopsy (Melzer et al., 2008; Patel et al., 2018b; Krieger et al., 2013; Stoianovici et al., 2016; Moreira et al., 2016), stereotactic neurosurgery (Sutherland et al., 2003; Nycz et al., 2017; Kim et al., 2017), and long bone biopsy (Cleary et al., 2018). On the other hand, patient-mounted robots are required to be compact, lightweight and easily attachable to the patient body. Some of the patient-mounted robotic systems for CT-guided percutaneous interventions which could potentially be adopted for shoulder arthrography are presented (Walsh et al., 2008; Maurin et al., 2008; Bricault et al., 2008); however, using CT results in exposure to radiation, and CT images have inferior soft tissue contrast. A robotic device for percutaneous interventions that can work both in the CT and MRI environments was presented by Hungr et al. (Hungr et al., 2016). An MRI coil-mounted, two degrees of freedom (DOFs) robot for cryoablation (Song et al., 2013) and a needle alignment mechanism for liver interventions were presented by Song et al. and Wu et al. (Wu et al., 2013), respectively. However, there are no lightweight and compact robotic systems suitable for pediatric patients. Our research groups have developed three generations of such robotic devices for shoulder arthrography procedures (Monfaredi et al., 2014; Patel et al., 2018a; Patel et al., 2019). A comprehensive review of MRI-guided robotic systems for needle-based interventions is presented in (Monfaredi et al., 2018).

In this paper we present a Thiel-embalmed cadaver study and clinical workflow studies of an integrated, patient-mounted robotic system for MRI-guided shoulder arthrography procedure in pediatric patients. This is a third generation system developed by our research groups and it improves upon previously reported robots (Monfaredi et al., 2014; Patel et al., 2018a; Patel et al., 2019). The robot attachment base is designed to provide robust and easy operation of the robot. In terms of maintaining a sterile field, the only part that needs to be sterilized is a needle stylet which can be easily inserted into the needle guide without any mechanical disassembly. The shoulder arthrography procedure was thought to be a good anatomical target for exploring the use of a body mounted robot, as respiratory motion is not an issue and there are few critical anatomical structures in the shoulder. In other work we are also investigating pain injections in the back and long bone biopsy under MRI guidance. Contributions of the presented work are 1) a kinematically identical but improved robot with covered cables for avoiding direct skin contact with the patient, 2) accuracy evaluation of an integrated system evaluation in Thiel-embalmed cadaver studies following entire clinical workflow, 3) improved shoulder-brace based robot attachment for improved robot attachment procedure compared to the strap-based mount used in the cadaver study, and 4) clinical workflow studies in healthy human participants for clinical usability of the system.

## 2 Methods

**FIGURE 1:**
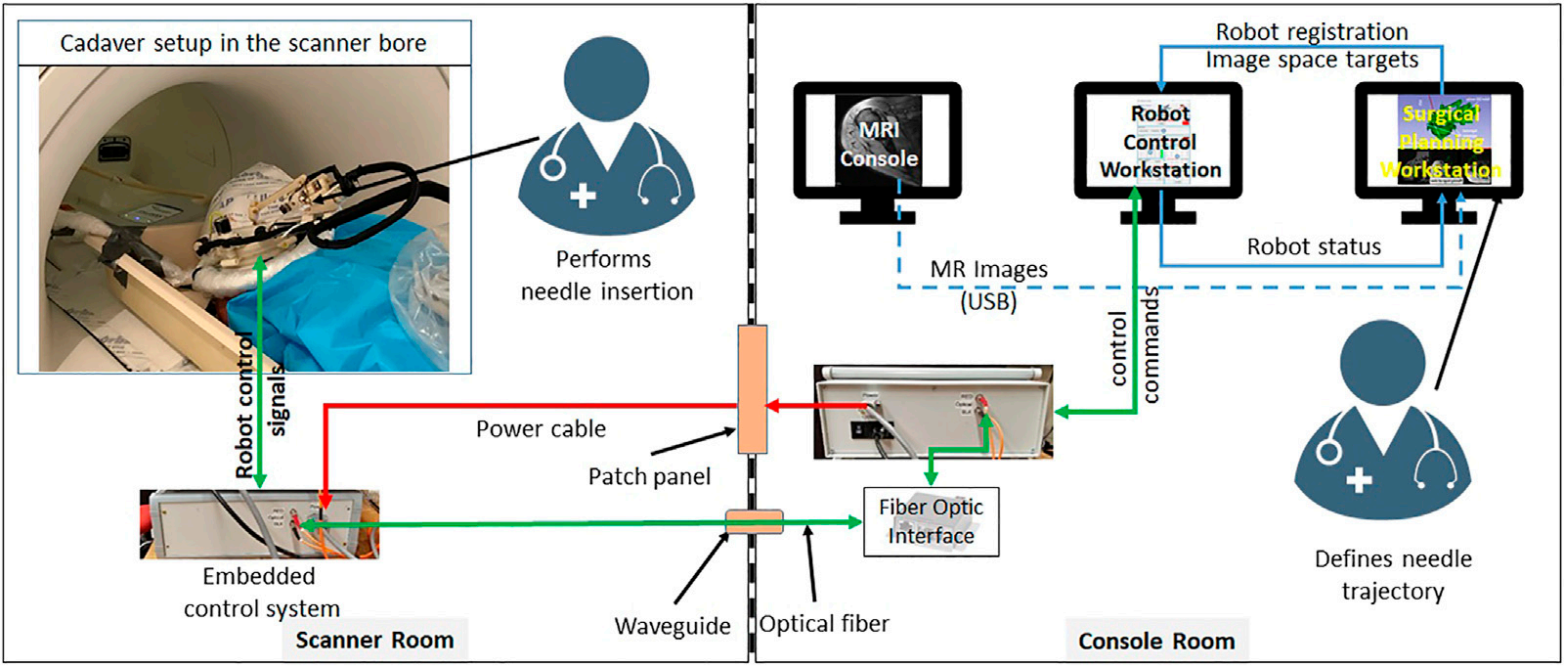
System block diagram showing all the components, their layout in the MRI facility and the data flow between them.

The integrated robotic system presented herein was evaluated in a Thiel-embalmed cadaver and in a workflow study with volunteers. Figure 1 shows the component diagram and the data flow between the system components.

### 2.1 Robotic Manipulator

**FIGURE 2:**
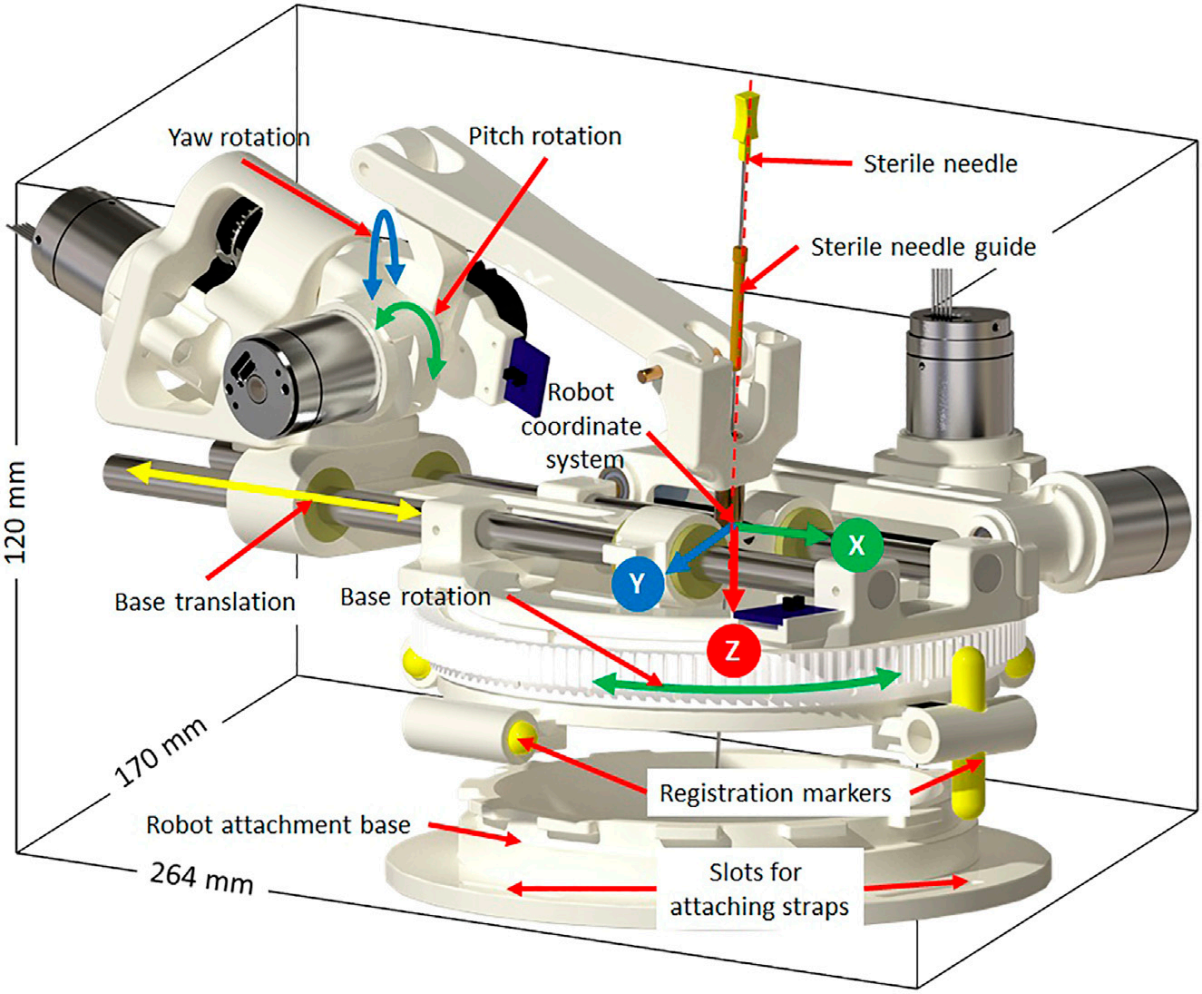
Robot CAD model showing the degrees of freedom, robot coordinate system and sterile stylet for needle insertion.

The robotic manipulator is a 4-DOF device with two DOFs for positioning and two DOFs for orienting the needle guide to a desired trajectory. The remaining two DOFs, needle insertion and rotation, are performed manually by the clinician. This manipulator preserves the kinematic structure of our previous robots (Monfaredi et al., 2014; Patel et al., 2018a), while being more rigid, accurate and optimized for clinical usage. It is a compact device with dimensions of 264 × 170 × 120 mm and weight of less than 700 g. All the components of the manipulator are manufactured from 3D-printed plastics and nonferrous materials such as aluminum and brass. Piezo LEGS ®(PiezoMotor, Upsala, Sweden) motors are used for actuation, while quadrature encoded differential optical encoders (E4T, USDigital, Vancouver, WA, United States) with 2000 counts per revolution (CPR) resolution provide precise yet relative joint positions: as the encoders provide relative position, each joint has an opto-interrupter (RPI-221, ROHM Semiconductor, Kyoto, Japan) based optical limit switch to define absolute position offsets. Using the limit switches, the robot is first calibrated and then initialized by moving each joint to the limit switch and then setting the joint position to a known offset. All four joints of the robot are controlled by a four-axis motion controller (Galil DMC 4143, Galil Motion Control, Rocklin, CA, United States) producing control signals for the piezoelectric motor drivers. Figure 2 shows a CAD model of the robot with all the components and coordinate systems. As shown in Figure 1, the embedded control system is placed inside the MRI room and connects to the robot using a 20 ft long cable allowing movement of the robot in/out the MRI scanner bore. The control system gets power supply via a DB-9 connector on the MRI room patch panel to avoid any radio frequency noise from entering the MRI room and affecting the image quality. The robot control application and an embedded control system communicates using a fiber optic cable passed through the wave guide on the patch panel of the MRI room.

Sterility is an important aspect when such a system is being used in clinical environment. The design of the robot mechanism is optimized to maintain the sterile field without affecting the clinical workflow. The needle guide is the only component that comes in direct contact with the injection needle while performing the insertion. As shown in Figure 2, the needle guide is designed such that first a sterile brass stylet is inserted to create a barrier between the sterile and non-sterile environments and then a sterile needle can be inserted.

We evaluated this robot in bench-top setting (Patel et al., 2018a) and anthropomorphic phantom studies (Patel et al., 2019) and achieved needle placement accuracy of 1.92 mm and 1.28° at the needle tip, which motivated the cadaver study presented herein.

### 2.2 Cadaver Study

**FIGURE 3:**
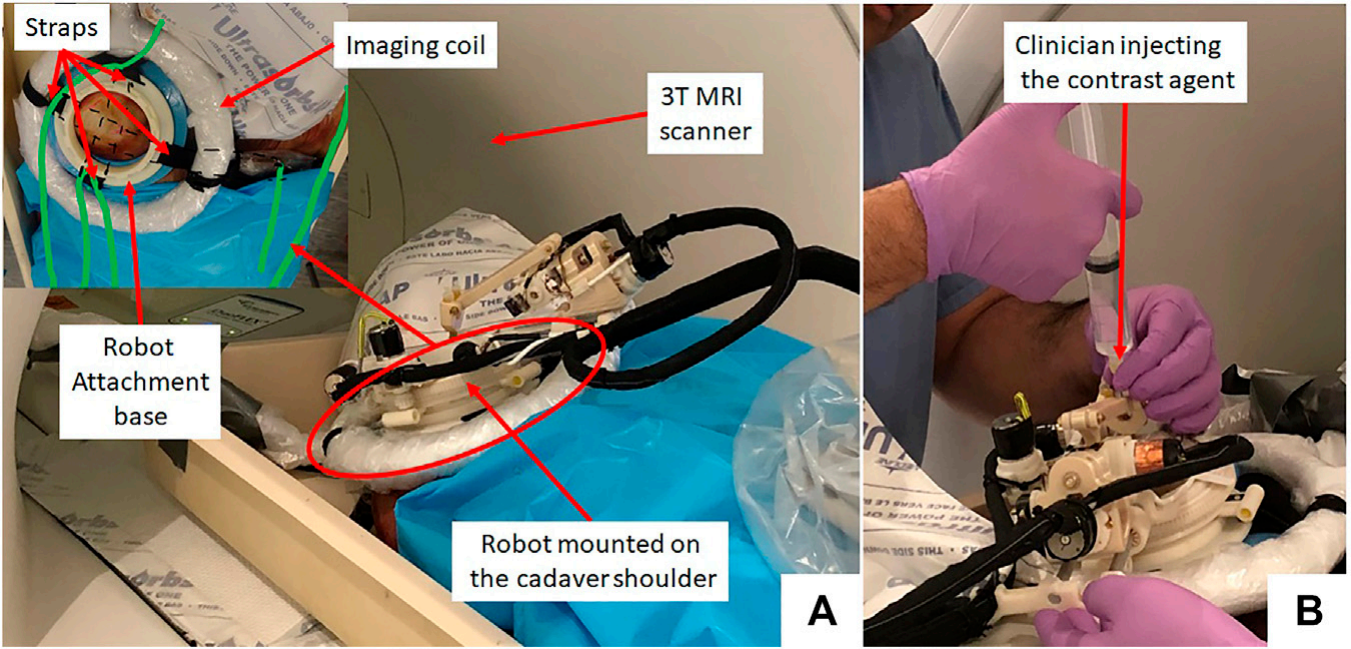
**(A)** Cadaver study setup showing the robot attached on the shoulder using straps and **(B)** a clinician injecting the contrast agent.

Thiel-embalmed cadavers were prepared at the Center for Anatomy and Human Identification (CAHID) and transferred to Institute for Medical Science and Technology (IMSaT), University of Dundee, United Kingdom. The cadaveric research was conducted in compliance with relevant anatomical legislation and local ethics and all donors having given their consent in accordance with the Anatomy Act (1984) and the Human Tissue (Scotland) Act (2006). Thiel-embalmed cadavers (Thiel (2002)) offer significant advantages over traditional formalin-embalmed and fresh cadavers including improved tissue flexibility, texture and tone, and low infection risk and odor. Thiel-embalmed cadavers retain vascular patency resulting in cadavers that can be perfused and imaged in multiple imaging modalities, which yields anatomically realistic preclinical models (Gueorguieva et al., 2014). Imaging was performed with a 1.5 T GE Signa HDx scanner (GE, Milwaukee, United States of America) using a eight channel DuoFLEX phased array coil (4CH, 24 cm paddle combined with 1CH interventional loop coil) at the IMSaT. As shown in Figure 3, a mounting ring was attached on the shoulder of the cadaver using straps with the loop coil around the mounting ring and square paddle underneath the cadaver. The goal of this study was to evaluate the needle placement accuracy of the robotic system *in vivo* while following the clinical workflow. Approval for the study was obtained by our collaborators at IMSaT. A total of 10 locations in the glenohumeral intra-articular space were targeted with different skin entry points to demonstrate that the robot could align the needle to any desired oblique trajectories and avoid any critical structures or bone collisions on the needle path.

**FIGURE 4:**
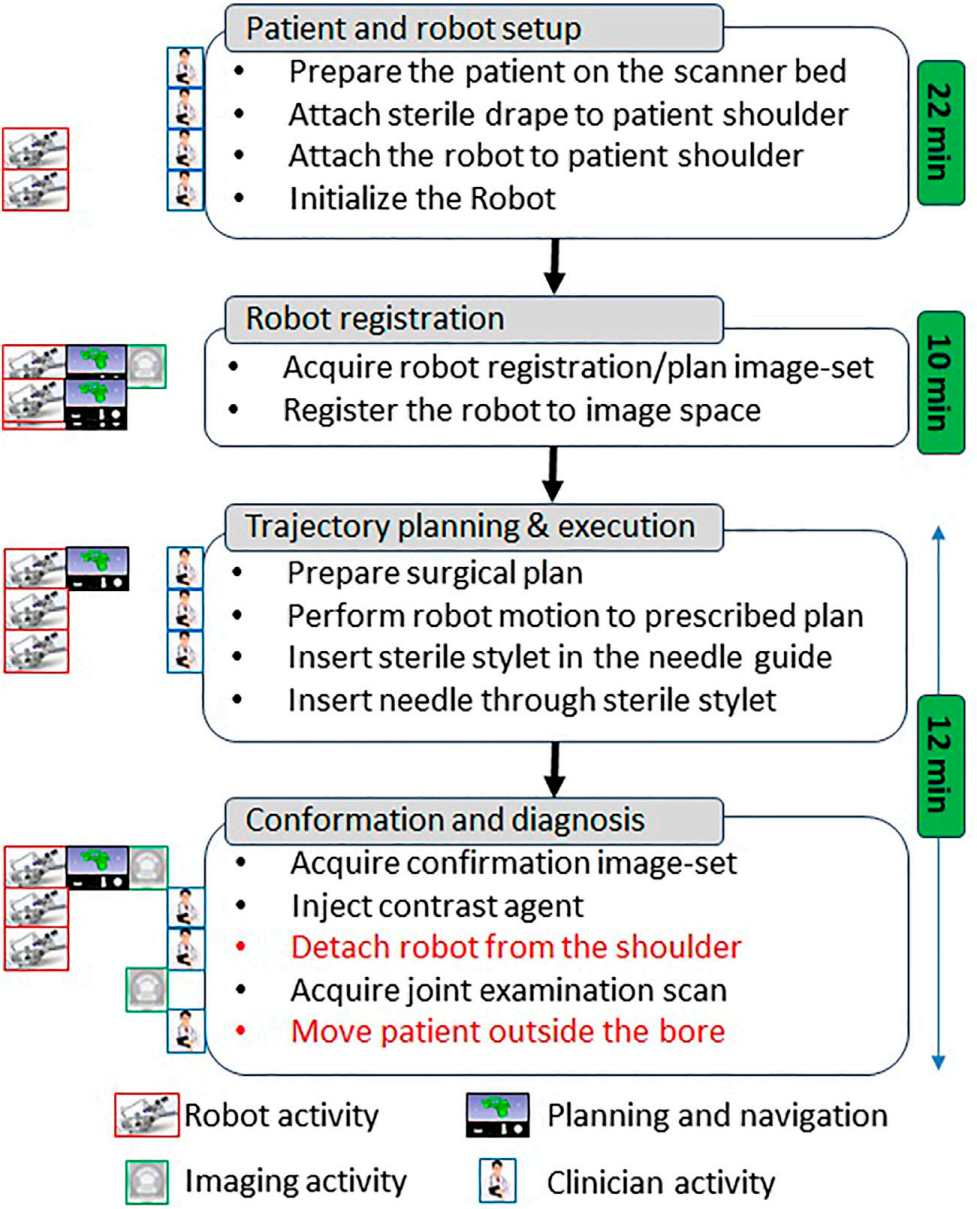
Proposed clinical workflow showing various steps for the robot-assisted, MRI-guided shoulder arthrography procedure. Procedure is divided into four phases, at the left of each activity shows what/who are involved for that activity, while for each phase measured average time is noted on the right. Duration for activities shown in red is not considered for the presented cadaver study as multiple targeting attempts were made in the same session.

**FIGURE 5:**
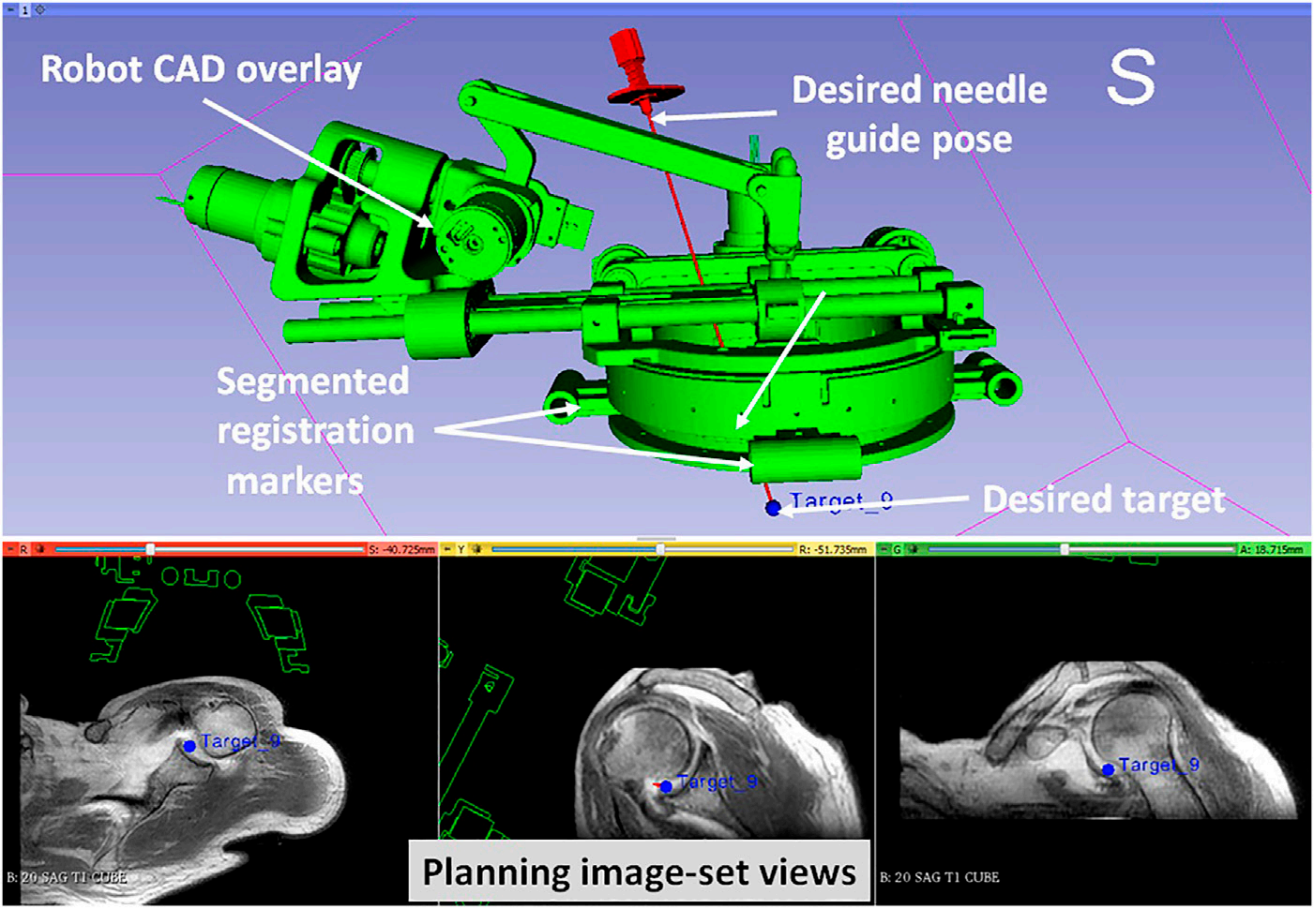
3D Slicer interface showing planning and navigation information, also shows four fiducial markers for registering robot to the scanner coordinate system

The clinical workflow followed for this cadaver study is shown in Figure 4 with the average duration for each phase measured during the cadaver study. The workflow was a four phase process: 1) cadaver and robot setup, 2) robot registration, 3) trajectory planning and robotic alignment (Figure 5) and 4) trajectory confirmation and contrast agent injection. Figure 3A shows the cadaver on the scanner table and robot attached to the shoulder while Figure 3B shows the clinician injecting the contrast agent. Following is the list of workflow steps followed during the cadaver study with status of the robot being powered ON/OFF shown in the brackets at the end of each step; though the robot does not cause any image artifacts (Patel et al., 2018a), it is powered OFF during imaging to ensure that there is no radio frequency interference from the robot control system:

1. Place the cadaver on the scanner bed in supine position (OFF);
2. Attach the robot attachment base with straps (OFF);
3. Attach the robot to the strapped mount (OFF);
4. Initialize the robot by moving each joint to the limit switched and then to the home positions (ON);
5. Move the scanner table to the isocenter (OFF);
6. Acquire Planning/Registration Image Set (OFF);
7. Perform robot registration and send it to robot control application using OpenIGTLink protocol (OFF);
8. Define needle trajectory by selecting target and entry points using 3D Slicer interface as shown in Figure 5 (OFF);
9. Send the planned target/entry points to robot control application over OpenIGTLInk (OFF);
10. Move the scanner table outside the scanner bore (OFF);
11. Move the robot to align the needle guide to planned trajectory (ON);
12. Move the table back to isocenter and acquire confirmation image set (OFF);
13. Confirm needle placement and move the cadaver out of the bore (OFF);
14. Inject the contrast agent (saline) (OFF);
15. Move the scanner table back to the isocenter and acquire diagnostic image set (OFF);
16. Repeat Steps 8–15 for Each Target.

### 2.3 Clinical Workflow Studies

**FIGURE 6:**
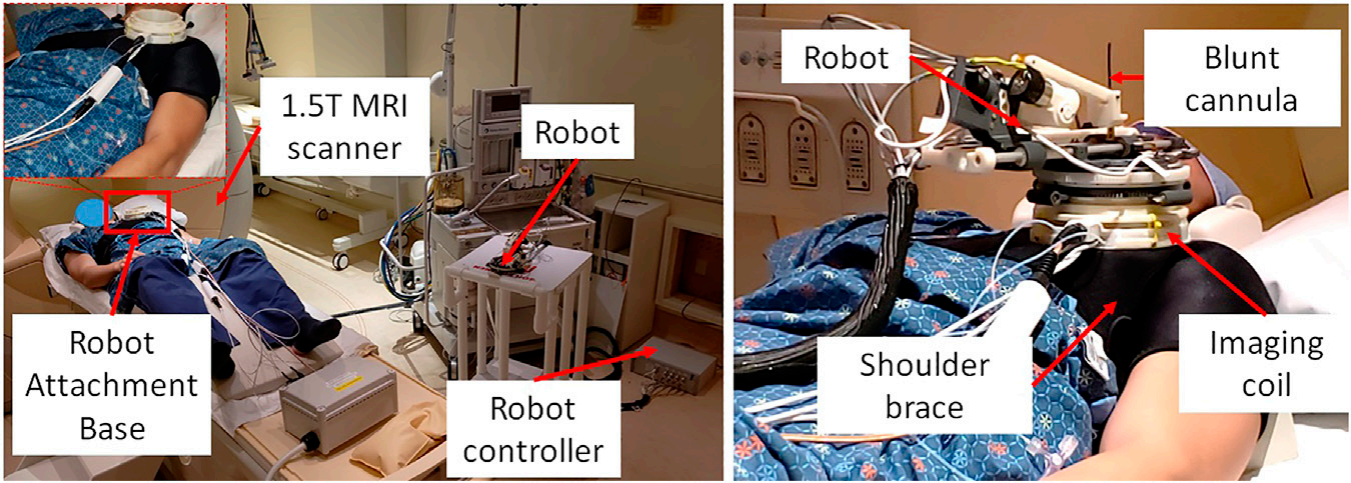
System setup for one of the volunteer study showing **(left)** placement of system components in an interventional MRI suite and **(right)** robot attached on the shoulder of the volunteer using a shoulder brace.

During the cadaver study, the entire clinical workflow was performed only once without attaching/detaching the robot between multiple targeting attempts. To better evaluate the system usability with varying patient anatomy, we conducted a multi-volunteer study under IRB approval at the Children’s National Hospital. The aim of this study was to evaluate the system usability in healthy volunteers, following the entire clinical workflow except the needle insertion and contrast agent injection. Also, during the cadaver study, we learned that the strap based robot attachment system was not effective, hence we updated it with a shoulder brace and an integrated single loop coil (Monfaredi et al., 2019) for better image quality. Robot was attached on the volunteer shoulder using a shoulder brace with the robot attachment base as shown in Figure 6. As shown in Figure 6, the integrated system was setup in an interventional MRI suite with Philips Achieva 1.5 T scanner and five healthy volunteers (3 males and two females) were recruited for a total of eight workflow studies. During each of the studies, the clinical workflow depicted in Figure 4 was followed, however, as these were healthy volunteers, the needle insertion step was performed using a blunt cannula inserted through the needle guide but not contacting the skin of the volunteer. Although, neither needles could be inserted nor contrast agent injected during these studies, we did a confirmation MRI scan to measure the total procedure time.

## 3 Experiments and Results

### 3.1 Cadaver Study

On a female cadaver, a total of 10 targeting attempts were made in the right glenohumeral joint. Robot was registered to the scanner coordinate system for the first targeting attempt and then the same registration was used for remaining targets. For each target, the clinician defined target and entry locations by clicking on the 2D slice views on 3D slicer. Robot registration transform, planned target and entry locations were sent to a MATLAB (The MathWorks Inc., MA United States) based robot control application using OpenIGTLink protocol. Robot control application calculated the desired joint positions using the inverse kinematics (Monfaredi et al., 2014). Desired joint positions were sent to an embedded control system which commanded all the joints to desired position under closed-loop position control. Once the needle guide was robotically aligned to the desired needle trajectory, the clinician manually inserted the needle to calculated insertion depth shown on the robot control application. After each needle insertion, needle position was confirmed with an intraopertive MRI scan (Proton Density-Turbo Spin Echo, TR/TE = 2,600 ms/42 ms; Flip angle = 150°; Slice thickness = 3; Pixel spacing = 0.78 × 0.78 mm; Scan time = 180 s). After clinician visually confirmed that the needle tip was in the glenohumeral intra-articular space, contrast agent was injected for the first targeting attempt to demonstrate the complete clinical workflow. All the targeting attempts were successful. However, fFor the first targeting attempt, the total time was more than 90 min as it involved the complete clinical workflow including cadaver preparation, robot attachment, registration to the scanner coordinate system, trajectory planning and contrast agent injection. For the remaining nine targeting attempts, the average time was 12 min which included only trajectory planning, robot motion and needle position confirmation. Figure 3 shows the cadaver with the robot attached to the shoulder using the straps and the clinician injecting the contrast agent after needle is precisely inserted into the intra-articular space of the glenohumeral joint.

**FIGURE 7:**
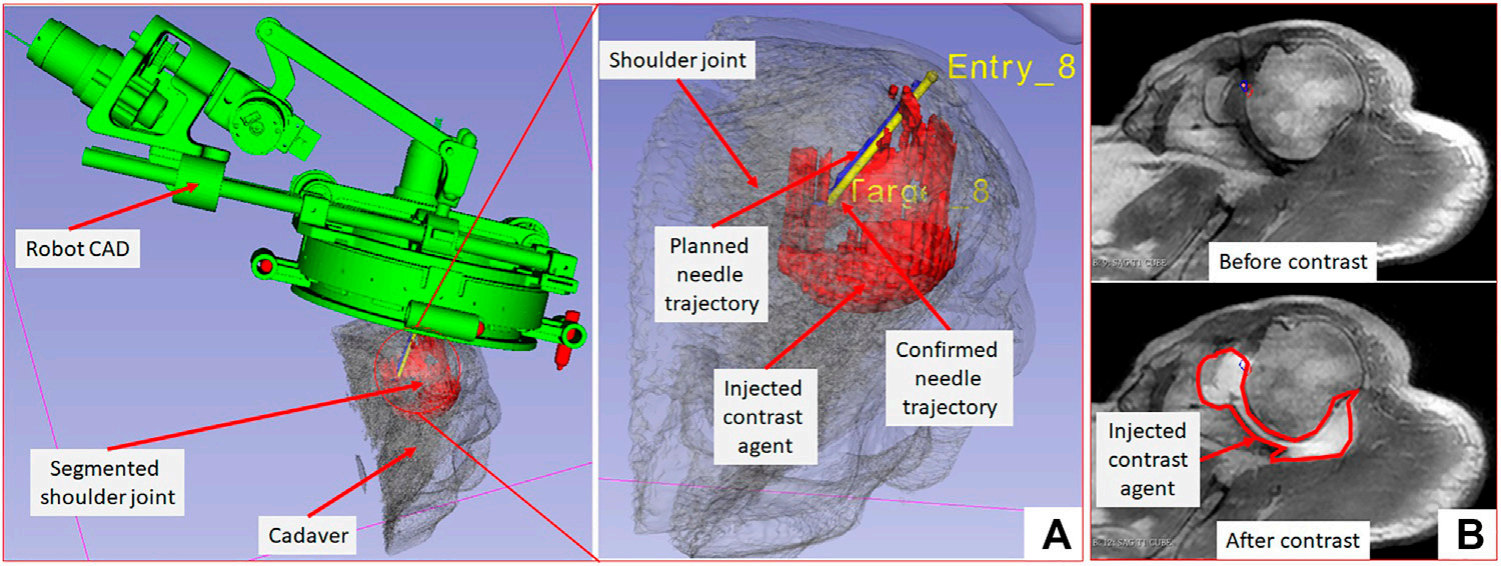
3D Slicer scene showing segmented 3D volume of the glenohumeral joint, injected contrast agent, target and entry locations, and planned and achieved needle trajectories.

The needle placement accuracy was assessed by manually selecting more than 40 points on the confirmation images following the needle artifact. To calculate the achieved needle pose, a line was fitted on those selected points. The needle tip placement error was measured as the minimum distance between the desired target location and the needle trajectory, hence eliminating any error along the insertion direction which was performed manually. The orientation errors are represented as the Euler angles between the measured and planned needle poses. Figure 7A shows one of the targeting attempts with the planned and confirmed needle trajectories along with the segmented glenohumeral joint and injected contrast agent, while Figure 7B shows one of the MR images of the glenohumeral joint before and after injecting the saline solution in the intra-articular space. As shown in Table 1, the robotic system has average needle tip positioning errors of 1.90, 0.65, and 2.17 mm in R (Right), A (Anterior) and S (Superior) directions respectively, and average needle pose orientation errors of 1.27° and 0.61° about R and A axis respectively. Errors for rotation about the S axis represent needle rotation and since this rotation cannot be measured from the needle artifact in MR images, they are not presented. Also, the position error along the S axis represents human error as needle insertion was performed manually and could be improved with motorize insertion. The average in-plane (except in the insertion direction and needle rotation about its axis) errors were 2.07 ± 1.22 mm and 1.46 ± 1.06 mm. The average residual error for the registration frame used in this robot is 1.2 ± 1.4 mm and contributes to the targeting errors.

**TABLE 1:** Results from 10 targeting attempts showing position and orientation errors.

Target no	Planned target			||Error||				
	R (mm)	A (mm)	S (mm)	R (mm)	A (mm)	S (mm)	R_*R*_ (deg)	R_*A*_ (deg)
1	−46.90	17.32	−35.00	2.15	0.25	0.08	0.60	0.34
2	−48.20	19.35	−38.23	0.39	0.10	0.53	1.28	0.19
3	−42.96	18.89	−29.54	0.85	0.40	3.88	0.28	0.17
4	−46.61	18.74	−35.73	2.76	0.56	1.83	2.76	0.58
5	−43.51	17.93	−25.23	0.80	1.08	2.00	0.21	0.12
6	−40.97	19.18	−27.50	1.65	0.27	1.95	2.13	0.31
7	−41.68	18.34	−30.50	4.53	0.65	1.01	2.29	2.23
8	−41.63	23.37	−30.79	1.36	0.21	3.90	0.52	0.37
9	−44.81	22.18	−34.42	1.85	1.07	1.13	0.94	0.44
10	−51.73	18.71	−40.73	2.63	1.89	5.36	1.66	1.37
Mean				1.90	0.65	2.17	1.27	0.61
STD				1.21	0.55	1.69	0.91	0.67

### 3.2 Clinical Workflow Studies

**FIGURE 8:**
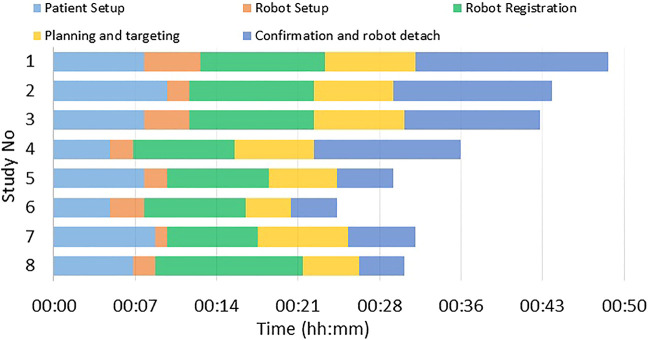
Results from the clinical workflow studies showing duration for each of the workflow steps.

A total of five healthy volunteers (3 male and 2 female) were recruited for these studies. For each of the study, the robot was attached on the shoulder of the volunteer using the shoulder brace mount shown in Figure 6 and entire clinical workflow depicted in Figure 4 was performed except the needle insertion and contrast agent injection. We recorded the duration for each of the workflow steps and results are show in Figure 8. The average duration for the entire workflow for these studies was 36 min. As the purpose of the volunteer studies was to evaluate the feasibility of the clinical workflow, though the robot was moved to the planned needle trajectory, needle placement accuracy was not evaluated.

## 4 Discussion and Conclusions

In this paper we reported a Thiel-embalmed cadaver study and multi-volunteer feasibility study under intraoperative MRI guidance for performing shoulder arthrography using a robotic system. The Thiel-embalmed cadaver is an excellent anatomy model for technology and clinical workflow evaluation and provides useful data that will be important as we move to clinical studies. The needle placement accuracy for injecting a contrast agent while following a clinically relevant workflow was evaluated, and duration for each of the workflow step was measured. The robot itself was an improved version of previously reported manipulators (Monfaredi et al., 2014; Patel et al., 2018a); various components such as the robot attachment base, sterile needle guide and clinical workflow were improved over previous systems to make it suitable for clinical usage. One of the shortcomings of this robot is that it is a serial mechanism so robot motion causes undesired cable movement; we improved cabling by covering all the conductive materials in a plastic wrap to improve patient safety and reduced cable weight by using thinner cables to lessen any undesired robot deformation due to cable weight. Also, during the cadaver study, we found it difficult to mount the robot because the shoulder of the cadaver was smaller than the robot attachment base (Figure 5), we used soft foam pads underneath the robot attachment base to stabilize it. This issue was resolved by using a shoulder brace during the volunteer studies by facilitating better contact between the bony anatomy and the mounting plate.

The cadaver study showed the feasibility of using the robotic device for accurate needle placement under intraoperative MRI guidance. Achieved needle placement accuracy of 2.07 mm with the robotic assistance is better than previously reported manual (3.1 ± 1.2 mm) (Fritz et al., 2012), robot-assisted abdominal interventions (4.1 mm) (Ghelfi et al., 2018) and liver interventions (4.1 ± 3.1 mm) (Hata et al., 2016). Though the accuracy improvement is not huge, robotic system can deliver this performance irrespective of the surgeon’s experience. Also, the robotic assistance allows needle insertions at oblique trajectories and potentially avoid any critical structures. With the achieved needle placement accuracy of 2.1 mm in this cadaver study, the needle was placed inside the glenohumeral intra-articular space with 100% (10 of 10) success rate. Some of the factors contributing to the reported errors are mechanical accuracy of the robot mechanism, robot-scanner registration error and needle deflection due to needle-tissue interactions. Moreover, the clinical workflow studies with multiple male/female volunteer showed that the system could be used on patients with varying shoulder size and entire workflow could be finished in under 1 h; it could be seen in Figure 8 that the total time for the procedure got shorter as the team got more experience with the system. However, during these studies we identified issues that must be resolved before moving to clinical trials: 1) robot cable management needs to be improved to ensure that the cable does not move during the procedure as it lays on the patient body, 2) identify and explore ways to drape the robot to maintain sterile field, and 3) sterilization of the parts that come in contact with the injection needle. Our future work will focus on improving those aspects of the system and evaluating it in clinical studies. Also, during the clinical studies we will evaluate the impact of patient motion on the targeting accuracy.

## Data Availability

The original contributions presented in the study are included in the article/Supplementary Material, further inquiries can be directed to the corresponding author/s.
